# Electrically-tunable positioning of topological defects in liquid crystals

**DOI:** 10.1038/s41467-020-16059-1

**Published:** 2020-05-05

**Authors:** John J. Sandford O’Neill, Patrick S. Salter, Martin J. Booth, Steve J. Elston, Stephen M. Morris

**Affiliations:** Department of Engineering Science, University of Oxford, Parks Road, Oxford, OX1 3PJ UK

**Keywords:** Liquid crystals, Polymers, Laser material processing, Liquid crystals, Topological defects

## Abstract

Topological defects are a consequence of broken symmetry in ordered systems and are important for understanding a wide variety of phenomena in physics. In liquid crystals (LCs), defects exist as points of discontinuous order in the vector field that describes the average orientation of the molecules in space and are crucial for explaining the fundamental behaviour and properties of these mesophases. Recently, LC defects have also been explored from the perspective of technological applications including self-assembly of nanomaterials, optical-vortex generation and in tunable plasmonic metamaterials. Here, we demonstrate the fabrication and stabilisation of electrically-tunable defects in an LC device using two-photon polymerisation and explore the dynamic behaviour of defects when confined by polymer structures laser-written in topologically discontinuous states. We anticipate that our defect fabrication technique will enable the realisation of tunable, 3D, reconfigurable LC templates towards nanoparticle self-assembly, tunable metamaterials and next-generation spatial light modulators for light-shaping.

## Abstract (web summary)

Defects in liquid crystals (LCs) find application in self-assembly of nanomaterials, optical-vortex generation and in tunable plasmonic metamaterials but methods for flexible and tailored fabrication of defects are scarce. Here, the authors demonstrate fabrication and stabilisation of electrically-tunable defects in an LC device using two-photon polymerisation, revealing interesting electro-optic behaviour.

## Introduction

Topological defects have long been of interest to theoreticians and experimentalists alike due to their ubiquity in ordered physical systems and more-recently due to their emerging use in practical applications. In condensed matter physics, topological considerations play a crucial role in explaining the properties of a wide range of materials including superfluids^1^, superconductors^2^ and topological insulators^3^. In particle physics and cosmology, topological defects have been studied as a candidate for explaining the density-fluctuations in the early universe that led to structure-formation and consequently, the existence of galaxies, stars and planets^4^. It has been crucial to the development of understanding in the topology of materials that the mathematics used to describe the formation and evolution of topological defects can be transferred across different length-scales and between different disciplines. To this end, defects in liquid crystals (LCs) have been investigated as highly-analogous laboratory systems for studying both superconductivity^2^ and the evolution of the early-universe^5^.

Nematic LCs are rod-like anisotropic organic molecules that display intriguing self-organising behaviour where there is long-range orientational ordering of the long axes of the molecules. The average local orientation is described mathematically by a unit vector called the director, **n**. Self-organisation and defect-formation in LCs is governed by the free-energy density associated with the possible arrangements of the anisotropic molecules in space^6^. In certain cases, LCs can self-organise into structures which contain singularities in the director field that describes the orientation of the LC director in space. Such a situation can occur due to the strong surface-anchoring of the LC director adjacent to a surface, establishing boundary conditions that make it impossible to have a continuous director field throughout the LC. In this study, we employ direct laser writing to fabricate 3-dimensional alignment surfaces that stabilise topologically discontinuous states and defects within a LC device. We also demonstrate subsequent tunability and control of the defects via an applied voltage and use this phenomenon to transport microparticles doped into the LC system.

Defects in LCs are of interest both from a fundamental perspective and due to their potential in a multitude of applications^7^. It has been observed that nanoparticle inclusions tend to migrate to defects in LCs as this minimises the overall elastic energy of a LC-colloid composite system. Consequently, topological defect networks have been proposed as templates for the self-assembly of nanoparticles^8–11^, allowing intricate structure formation without the need for top-down lithographic approaches. Other design-modes are also possible by changing the nature of the colloidal particles. For example, by doping an LC defect system with self-propelled swimming bacteria, it has been found that the presence of defects controls and influences the normally-chaotic swimming behaviour of the bacteria^12^. Furthermore, the use of plasmonic or metallic particles as colloidal dopants in LCs has led to the exploration of tunable metamaterials and plasmonic materials^13–15^. An emerging area of LC defect research is in advanced spatial modulators^16^ for controlling polarisation and optical fields via the Pancharatnam-Berry Phase^17,18^. Crucially, for all of these applications, reliable methods of controllably generating and stabilising defects must be developed to enable the engineering of functional defect patterns and networks. Recently reported methods have typically been limited to substrate patterning, usually with photoalignment techniques^19^ or by employing electrically-induced nematic flows^20^. Using these methods, the creation of large-scale arrays of defect lines has been demonstrated^21^ but these have mostly been static networks with a limited degree of tunability. Our approach allows the arbitrary generation, stabilisation and electrical-control of defects in a precise, single-step process over a large area.

Two-Photon Polymerisation Direct Laser Writing (2PP-DLW) has emerged as a powerful laser-processing technique that can be used to fabricate polymer structures on the micro/nanoscale^22–24^. High intensity ultrafast pulses from a femtosecond laser are focused into a polymerisable resin, whereupon the light is absorbed by photoinitiators that trigger a free-radical polymerisation process, crosslinking the monomers in the resin. It offers an advantage compared to conventional focussed UV laser/LED systems because the absorption occurs in a smaller volume (a “voxel”) due to the nonlinear light-matter interaction that occurs as a result of the high intensity in each laser pulse. By translating the sample with respect to the focus of the laser, fine structures can be fabricated in 3D, with the possibility of achieving a resolution below the optical diffraction limit^25,26^.

Typically, the photoresists used in 2PP-DLW form isotropic polymers where there is no molecular ordering to the crosslinked polymer chains and the functionality of the materials is controlled solely by the micro/nanostructure of the 3D design. Our resin is a liquid crystalline mixture, allowing us to tune the properties of the resin itself by altering the molecular alignment and order with external fields during fabrication^27,28^. Polymerisation of the resin creates polymer features with a controllable order/alignment, which function as 3D alignment surfaces for neighbouring LC molecules. Using this method, different molecular alignments and topologies can be polymerised in the same device with resolution on the microscale. We exploit this capability to stabilise topologically discontinuous states and disclination lines inside an LC device. Furthermore, by carefully tailoring the shape of the polymer structures, it is possible to pre-configure/program the motion of the disclination lines that move when the voltage applied to the device is altered. Thereby bringing order to the usually-random and disordered process of defect nucleation and growth in LC devices.

## Results

### Topologically discontinuous states in liquid crystal pi-cells

In this study we use LC pi-cell devices, which enable electrical-addressing of a layer of nematic LC aligned between parallel-rubbed glass substrates^29^. We use such glass cells for this demonstration because, in contrast with anti-parallel-rubbed cells, it is possible to induce different topologies in the LC director by applying a low-voltage electric field across the device to generate molecular alignments that are topologically distinct. The pi-cell consists of two glass substrates coated with parallel-rubbed alignment layers, sandwiching a layer of LC a few microns thick. In the ground state, with no applied voltage, the LC director adopts a homogeneous splay state (“H-state”) across the device (Supplementary Fig. 1a). Applying a voltage initially causes the alignment to distort, forming first an asymmetric H-state (“H_a_-state”) and, as the voltage is increased above a critical voltage, V_c_, the bend state (“V-state”) (Supplementary Fig. 1a). The V-state is topologically distinct from the H-states and grows slowly after nucleation, forming a domain that is bounded by a strength ± ½ disclination loop, as long as the voltage remains above V_c_^30^. If the voltage is reduced below V_c_, the H-state nucleates and grows, slowly consuming the entire device via the discontinuous transition back to the ground state.

Reducing the voltage to <~V_c_/2 results in the bend-state collapsing to a 180° twisted state (“T-state”) (Supplementary Fig. 1a). However, at any voltage below V_c_, the H-state will grow, slowly consuming the entire device via the discontinuous transition back to the ground state from either the T or V states. The complete-picture of pi-cell behaviour is evidently complex, but it is only important in this study to note that the V and T states are topologically distinct from the H-states and thus can only exist in the same device with a disclination line separating the domains. Furthermore, it should be noted that the nucleation and growth of the topologically discontinuous states in a conventional pi-cell is a random process (see Supplementary Fig. 2) and this is a key factor preventing pi-cells from finding use in applications^30^. All of these topologically distinct director states and the disclination lines themselves can be observed and identified in a straightforward manner with a polarising optical microscope.

### Electrically-tunable disclination line

**Fig. 1:**
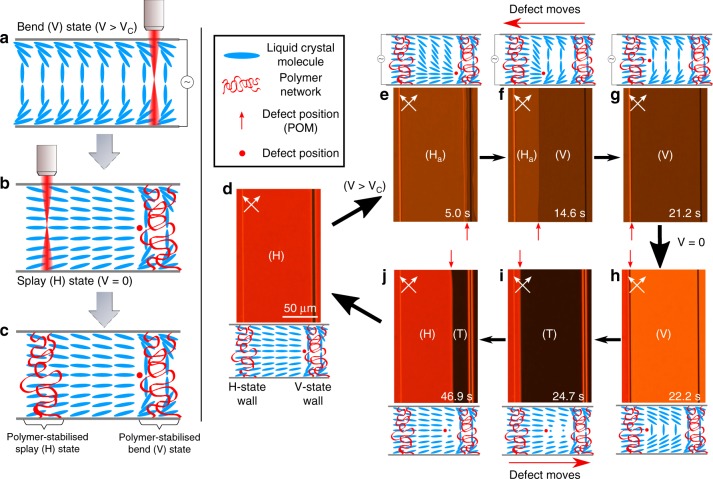
Electrically-tunable disclination line. Left: Schematic of the fabrication procedure showing a cross-section of the LC device. Blue rods show the orientation of liquid crystal molecules, red circles indicate the defect position and red ribbons represent polymer network. **a** Initially the device is driven into the bend state (V-state) by applying a voltage above V_c_, before 2PP-DLW is used to create a polymer wall stabilised in the V-state. **b** The applied voltage is removed and the device relaxes to the splay state (H-state). A defect forms next to the V-state wall, separating the topologically discontinuous states. 2PP-DLW is then used to fabricate a polymer-stabilised H-state wall 100 µm from the first wall. **c** The final configuration of the device, with two parallel polymer-stabilised walls, in the topologically discontinuous H and V states, respectively, separated by a gap of 100 µm. Right: Polarising optical microscopy (POM) images and director profiles showing the dynamics of the tunable disclination line under an applied voltage. Red arrows highlight the position of the defect line in each POM image. **d** With no voltage applied, the bulk of the device is in the H-state and the defect is adjacent to the V-state wall. **e**–**g** A voltage above V_c_ is applied to the device and the defect begins to move across the channel until it meets the H-state wall. **h**–**j** Removing the voltage causes the V-state to relax into a 180° twisted state (T-state), and the defect moves back across the channel. The H-state grows and moves across the channel with the disclination line at the frontier of the growing domain, until it is impeded by the polymer-stabilised V-state wall.

The fabrication of a tunable disclination line is illustrated in Fig. 1, which shows schematic illustrations of the LC director and polarising optical microscopy (POM) images of the device under different applied voltages. By stabilising the splay (H) state and bend (V) state in a pair of parallel polymer walls, a disclination line can be moved across the gap between the walls with the application of a voltage across the LC device. The mixture used in this study consists of the nematic host, E7, that is doped with a reactive mesogen, RM257, and a photoinitiator (Irgacure 819) (see Methods section for further details). This mixture was filled into a pi-cell with a 5 µm gap between the substrates and placed in our bespoke direct laser writing system^28^. When the mixture is exposed to tightly-focussed femtosecond pulses from a Ti:Sa laser, a two-photon polymerisation process takes place, forming a polymer network and stabilising the alignment of the director at the moment of exposure. In this way, the tunable disclination line in Fig. 1 was fabricated in two steps: firstly, the V-state was stabilised by applying 10 V to the device before a 1 µm wide polymer wall was written by linearly translating the device under exposure to the laser (Fig. 1a). Subsequently, the voltage was switched-off and a polymer wall was fabricated in the H-state, parallel to the first wall and separated from it by 100 µm (Fig. 1b). The numerical aperture of the focussing lens for the fabrication laser was chosen such that polymer walls would be fabricated across the full thickness of the device. Scanning electron microscopy (SEM) was used to image laser-written polymer structures fabricated using our set-up in order to check that the polymer feature size and the size of the laser focal spot were closely matched (Supplementary Fig. 3). It was found that the features are 1 µm wide in good agreement with the laser spot size of the fabrication system thus indicating that the influence of molecular diffusion was minimal and the polymerisation terminates rapidly after photoinitiation.

As a result of the topological discontinuity of the two stabilised states, there is a disclination line adjacent to the polymer-stabilised V-state wall in the absence of an applied voltage (Fig. 1d). Application of a voltage that has an amplitude above the critical voltage required for formation of the bend state, V_c_, causes the defect to move as the V-state grows from the polymer-stabilised V-state wall. Due to the coupling between the voltage and the positive dielectric anisotropy of the liquid crystalline material, the V-state becomes the state with the lowest free-energy density and thus the V-state domain will grow, moving the disclination line at the frontier of the domain. The defect moves across the channel (Fig. 1f) until it reaches the H-state polymer wall, which halts the progress of the disclination. Switching-off the voltage causes the bend domain in the unpolymerised channel to collapse over a period of a few seconds (Fig. 1h) into a twisted state with a 180° twist (Fig. 1i). The lower energy H-state (the ground state of the system) then nucleates uniformly from the H-state polymer wall, moving the defect until it reaches the V-state wall (Fig. 1j). A real-time movie of this process can be seen in Supplementary Movie 1.

This relatively simple tunable-defect structure, consisting of an unpolymerised channel between polymer walls stabilised in different topologically discontinuous states can also be formed with other, less-sophisticated, fabrication techniques. Supplementary Fig. 4 presents results obtained using a simple UV LED photolithography system for single-photon initiated polymerisation to create a similar uncured channel between a polymerised H-state region and a V-state region. However, the nucleation of the V-state under an applied voltage is less uniform and predictable than in the structure fabricated with 2PP-DLW, as there are likely to be differences in the polymer network morphology along the edge of the polymerised region. Furthermore, the fabrication of more-complex structures that demonstrate advanced defect-control capability is considerably more challenging when using this photolithography technique, due to the need to perform multiple exposures with accurate registration between different photomasks. In addition, creating micron-scale features requires the projection of the photomask into the LC layer within the glass cell to prevent the shadowing effect that arises from placing a photomask on the surface of the outer substrates. We have also used a 405 nm laser in our direct laser writing system to trigger single-photon polymerisation, but found that there was widespread photo-polymerisation in undesired locations in the device due to scattering and back-reflection of the laser. As a result of these difficulties in using UV sources, we found 2PP-DLW to provide far greater resolution and precision when fabricating polymer networks embedded inside LC devices, so proceeded with this method for the stabilisation and electrical control of defects in this study.

### Disclination line control system

**Fig. 2:**
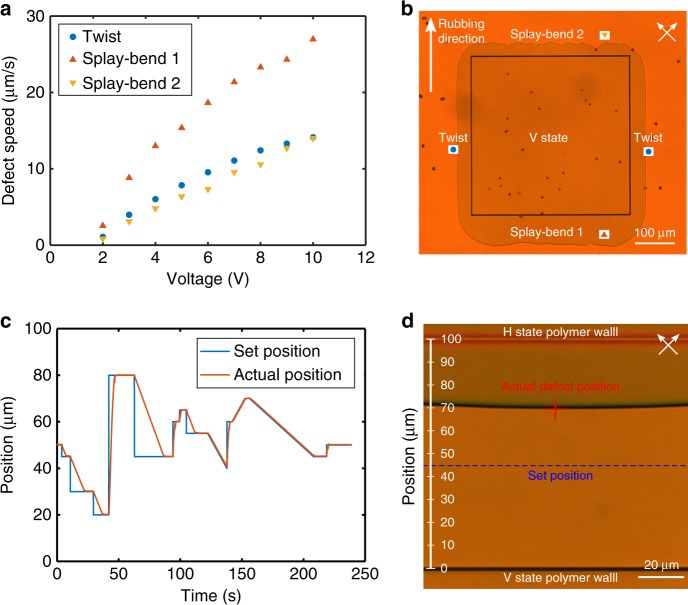
Tunable disclination line control. **a** Disclination line speeds as a function of voltage at 25 °C for different defect orientations with respect to the device rubbing direction. **b** POM image of the different types of defect that exist in LC pi-cells. A voltage V > V_c_ has been applied to the device, causing the V-state to grow and the defects to move away from the V-state polymer walls. Twist-type defects correspond to the case where disclination lines are parallel to the rubbing direction and splay-bend type defects exist when disclination lines are perpendicular to the rubbing direction. **c** A disclination line control system responding to an arbitrary series of changes in set position. **d** Snapshot POM image of the control system in operation. The set position is indicated with the dotted blue line and the red cross indicates the actual position of the defect as determined by the image recognition algorithm. The POM images **b** and **d** are between crossed polarisers and the devices are oriented such that the rubbing direction is 45° to the transmission axes of the polarisers.

The magnitude of the applied voltage influences the speed of the disclination line that moves when the V-state domain grows. At higher voltages, the defect travels faster, as the free-energy density of the V-state decreases with increasing voltage, making it energetically favourable for the state to grow at a faster rate. The relationship between disclination line speed and voltage can be seen in Fig. 2a for the different types of disclination line shown in the POM image in Fig. 2b. The twist-type defect is observed when the disclination line is parallel to the rubbing direction and is the type of defect shown in Fig. 1. Whereas, the splay-bend type defect occurs when the disclination line is perpendicular to the rubbing direction and is shown in more detail in Supplementary Fig. 5 and Supplementary Movie 2. Schematic director profiles of the two defect types are illustrated in Supplementary Figs. 1b and c. Due to the inherent asymmetry of the pi-cell that results from the parallel-rubbed substrates, it was observed that disclination lines oriented perpendicular to the device rubbing direction (splay-bend type) have a speed vs voltage relationship that depends on the direction in which they travel. This is in accordance with previously observed experimental^31^ and numerical studies^32^ of LC pi-cells that have shown that the dynamic behaviour is governed by anisotropic hydrodynamics and flow.

We have created a control system which demonstrates that it is possible to precisely control the position and speed of a tunable disclination line using a control loop to regulate the magnitude of the voltage applied to the device. Figure 2c shows the position of the disclination line in response to a series of changes in the set position. As can be seen in Fig. 2d, the tunable disclination line structure is identical to that shown in Fig. 1 and here positive changes in the set position correspond to the V-state growing under an applied voltage. Negative changes in set position correspond to the H-state growing and, as this is a relaxation process, the system responds more slowly in this direction. A schematic of the elements of the control system is illustrated in Supplementary Fig. 6a and a video of the system in operation can be seen in Supplementary Movie 3. The program receives a real-time video feed from a camera attached to a microscope imaging the LC device and performs image analysis to determine the position of the defect line. The position can be detected with an accuracy of ±1 µm, limited by the optical diffraction limit and the resolution of the camera. This measured position is compared with the set position to obtain an error signal. The voltage output from a function generator is made proportional to this error signal in order to move the defect to the desired position. When the error signal is zero (i.e. when the set position = actual position) an experimentally-determined hold voltage is applied to fix the disclination line at a precise position. A block diagram of this control system is shown in Supplementary Fig. 6b (also see the Methods section for further details). By implementing this feedback-based control, the position of a disclination line can be stabilised indefinitely and controlled programmatically.

### Tunable-defect-mediated microparticle transport

**Fig. 3:**
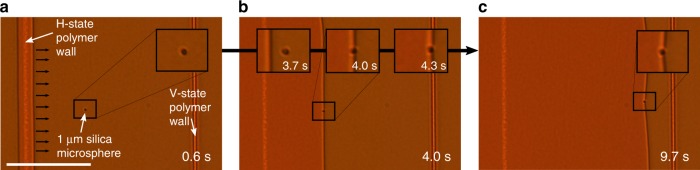
Disclination line cargo-transport. **a** A 1 µm silica microsphere is towed by a disclination line across the gap between topologically discontinuous polymer walls upon removal of a voltage V > V_c_ at *t* = 0. **b** When the disclination line encounters the microparticle it **c** carries it across the channel to the polymer-stabilised V-state wall. Scale bar 50 µm.

LC defect networks have been proposed as soft templates for the self-assembly of nano/microparticles. The reason for this is two-fold: (1) colloidal inclusions in LC hosts tend to become localised and trapped at defect cores and (2) LC mesophases have the ability to self-organise into complex macroscopic structures^8,11,33,34^. Furthermore, doping of colloidal particles into an LC host can result in new physical properties and functionality that are not intrinsic to the LC material. In addition, LCs can be employed as a tunable medium to influence the properties of the nano/microparticles^9,35^. Typically, the defect networks that have been employed in previous studies have been relatively static with a limited degree of tunability. In contrast, by doping our LC resin with functionalised 1 µm silica microspheres (see the Methods section for further details) we show here that the disclination lines can be used to pick up and re-position particles, as a proof-of-concept for disclination-mediated electrophoretic cargo-transport. Developing methods for micro/nanoparticle electrophoresis with AC driving schemes is of significant interest at present as it avoids unwanted electrochemical reactions that can occur using DC fields^36^. Figure 3 shows a microparticle between polymer-stabilised walls of different topology (identical to the structure in Fig. 1) being dragged by the disclination line across the 100 µm wide channel separating the polymer walls. The device, which is initially driven into the V-state with an applied voltage above V_c_, is switched-off at *t* = 0 (Fig. 3a), moving the defect from left to right. When the disclination line reaches the microparticle (Fig. 3b) it traps it and transports it across the channel (see Supplementary Movie 4).

### Topologically discontinuous polymer walls for engineered tunable disclination line control

Ordinarily, for a pi-cell, the topologically discontinuous states would nucleate randomly across the device with states forming approximately spherical domains that are bounded by strength ± ½ disclination loops (Supplementary Fig. 2). However, fabricating polymer walls using direct laser writing enables us to precisely control the motion of the defects in 2-dimensions. These tunable-defect structures are found to be completely reversible, with no hysteresis, and the velocity of the disclination lines has been shown to depend on the magnitude of the applied voltage and the orientation of the defect with respect to the rubbing direction.

**Fig. 4:**
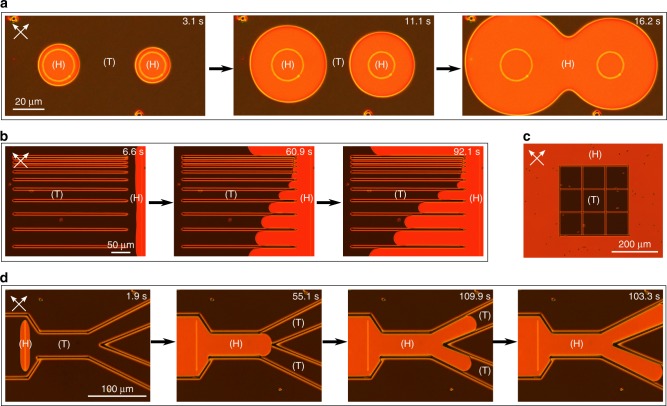
Complex polymer structures for tunable disclination line control. **a** Polarising optical microscopy (POM) images of circular disclination lines growing as the device relaxes to the ground state. **b** POM images of defect-confinement channels. Varying the spacing between adjacent laser-written V-state walls affects the speed of a defect passing through the channels. **c** POM image of a 100 µm pitch V-state polymer lattice in the absence of an applied voltage. The V-state polymer walls comprising the square lattice stabilise the twisted state, preventing the relaxation to the H-state within the square wells, even at zero voltage. **d** POM images of a microfluidics-inspired bifurcated defect channel. The walls defining the channel were laser-written in the V-state while, inside the channel, a wall was written in the H-state. When the voltage is switched off at *t* = 0, the H-state nucleates from this wall and a defect travels down the channels.

Using 2PP-DLW to precisely polymerise different topologically discontinuous states within the same device enables us to control this nucleation process and engineer the dynamic behaviour and morphology of the defect lines. To illustrate this, Fig. 4 presents examples of complex polymer wall designs that demonstrate further ways to control the dynamics of the defects via the design of the polymerised walls. Figure 4a shows circular defect rings formed by polymer walls laser-written in the H-state. When the device is driven to the V-state and subsequently switched-off, the defect will grow from the circular H-state polymer wall. When the defect lines meet, they annihilate, forming a continuous H-state domain (see Supplementary Movie 5). Figure 4b demonstrates that the speed of the disclination line is influenced by the degree to which the defect is confined by topologically discontinuous polymer walls. When the applied voltage is switched-off while the device is in the V-state, the H-state nucleates from the H-state polymer wall and the disclination line separating the topologically discontinuous states begins to travel across the device. When the disclination line meets the defect-confinement channels that were written in the V-state (structure design in Supplementary Fig. 7), the motion is impeded and the degree to which the defect slows-down depends on the width of the walls (see Supplementary Movie 6).

It is possible to permanently stabilise the V-state within a device over relatively large areas by forming enclosed square wells with 2PP-DLW (Fig. 4c). This is of technological interest because the V-state is the fast-switching state used in display applications of pi-cells and the slow transition from the H-state to the V-state leads to a very long warm-up time for such devices^37^. The device was driven into the V-state by applying a voltage above V_c_ and the polymer walls defining the 100 µm pitch square lattice were fabricated in this state. Upon removal of the voltage, the bulk of the device will relax slowly back to the H-state but the regions confined by the V-state polymer walls relax into the T-state and remain in this state indefinitely. Applying a voltage to the device causes the T-state to rapidly switch to the V-state, as there is no topological discontinuity between the two states. This result shows that it is possible to employ 2PP-DLW to engineer a pi-cell device without the slow H-to-V-state transition step, overcoming a fundamental limitation that has prevented pi-cells from finding technological applications.

Figure 4d shows a bifurcated channel structure inspired by recent developments in microfluidics technology. This represents a totally new approach to LC microfluidics where the ‘flow’ is a wave of director reorientation in the form of a propagating defect line, rather than conventional fluid-flow through a channel with microscale dimensions. The bifurcated channel is designed to guide the defect separating the H and V states as the device transitions between the states. Upon removal of an applied voltage that is above V_c_, where the device is in the V-state, the H-state nucleates from a polymer wall written in the H-state configuration before propagating down the channel. The H-state is unable to penetrate through the V-state polymer walls that define the channel, as they have been stabilised in a topologically discontinuous state (structure design in Supplementary Fig. 7). When the propagating disclination line separating the two states reaches the junction to the two separate channels, it is forced to move down these smaller channels (see Supplementary Movie 7). Using laser writing to create complex polymer structures such as this bifurcated channel allows the movement of defects in the device to be precisely controlled and localised to particular regions of the device.

## Discussion

In conclusion, we propose and demonstrate an approach to fabricating and controlling disclination lines with high-precision in LC devices, unlocking a broad gamut of electro-optic behaviour. Our platform of in-situ direct laser writing enables polymer structures to be fabricated directly inside electrically-addressable LC devices to lock-in voltage-dependent topologically discontinuous states. Remarkably, fabrication of these topological polymer structures introduces order and control to the normally-random and disordered nucleation processes that govern the behaviour of defects in the LC; something that is usually an unwanted phenomenon in LC technology that plagues the device performance. Here we show quite the reverse: that by harnessing these defects it is possible to observe a range of interesting phenomena such as microfluidics inspired flow of boundaries separating topologically discontinuous states without any actual material flow, the controlled transportation of microparticle suspensions with high precision, and the stabilisation of fast-switching states. The electrical tunability demonstrated via dynamic and reversible control of the disclination line position could play an important role in the realisation of tunable defect-scaffolds as templates for 3D self-assembly as well as the creation of artificial mesophases. Furthermore, this approach represents a paradigm-shift in LC device fabrication that could have far-reaching impact in emerging application areas of LC technology. In particular in the development of advanced spatial light modulators that use topological pixels to create structured light fields and introduce phase singularities in gaussian beams.

## Methods

### Preparation of polymerisable liquid crystal mixture

The polymerisable mixture used in this study consisted of the nematic LC mixture, E7 (70.7 wt%), reactive mesogen RM257 (Merck) (28.5 wt%) and photoinitiator Irgacure 819 (Merck) (0.8 wt%). The mixture was capillary filled in the isotropic phase into 5 µm thick pi-cells manufactured by Samsung with a pre-tilt of 4–6°. The cells consisted of two ITO coated glass slides, rubbed parallel and separated by 5 µm spherical spacer beads. After cooling to room temperature, the LC device was mounted in a custom-built 2PP-DLW system and connected to a function generator so that the voltage applied to the device could be varied during fabrication.

### Preparation of microparticle mixture for cargo-transport demonstration

0.9 µm diameter silica beads (Bangs Laboratories) were functionalised with the homeotropic alignment agent dimethyloctadecyl[3-(trimethoxysilyl)propyl]ammonium (DMOAP) (Sigma-Aldrich). DMOAP was supplied as a 42 wt% solution in methanol. Deionised water was then added and the methanol evaporated at 65 °C to form a 1 wt% solution of DMOAP in water. Thirty milligrams of the silica beads were added to 200 µL of the 1 wt% DMOAP solution and sonicated for 1 h at room temperature. The dispersion was then centrifuged at 3000 RPM for 5 min to sediment the particles. A pipette was used to extract the excess DMOAP solution, before deionised water was added to the vial. This process of centrifugation and replacement of the excess DMOAP solution with water was repeated five times. Finally, the water was evaporated at 105°C on a hotplate. A mixture was made of 5.5 wt% DMOAP-functionalised silica beads, E7 (Merck) (64.4 wt%), RM257 (Merck) (31.5 wt%) and Irgacure 819 (Merck) (1.5 wt%). This mixture was capillary filled into 5 µm thick pi-cells as described above.

### Two-photon polymerisation direct laser writing

Femtosecond laser pulses of duration 100 fs from a Spectra-Physics Mai-Tai titanium-sapphire oscillator emitting at 790 nm with a repetition rate of 80 MHz were focussed with a Zeiss 0.5 NA objective lens into the LC layer of the cell. The power of the fabrication laser after the objective was 23 mW. A Hamamatsu X10468-02 phase-only spatial light modulator was imaged onto the pupil plane of the objective lens in a 4 f configuration to correct for spherical aberrations introduced when focusing inside the LC device. Devices were mounted onto a stack of high-resolution air-bearing translation stages (Aerotech ABL1000) that allowed the sample to be moved relative to the laser focus with high precision and accuracy. A red LED was used to provide transmission illumination of the device so that the fabrication could be monitored in-situ with a monochrome CCD without affecting the photocuring process. Polymer walls were fabricated by moving the sample under continuous exposure to the pulsed laser beam at a speed of 100 µm/s. Polymer-stabilised bend-state walls were written with a 1 kHz AC square-wave voltage of 10 Vrms applied to the device and polymer-stabilised splay state walls were written at 0 V. SEM images of representative polymer structures can be seen in Supplementary Fig. 3.

### Optical microscopy

Microscopy was conducted using an Olympus BX51 polarising optical microscope with a QImaging Retiga R6 camera attached to the phototube. Olympus objective lenses were used with the cover slip correction collar set to the thickness of the glass slides comprising the LC device, to improve the quality of the images by reducing aberration. A long-pass filter with a cut-off wavelength of 550 nm was inserted between the halogen bulb and the sample to avoid causing any polymerisation in the samples that still contained unreacted reactive mesogen molecules. Devices were oriented such that the rubbing direction was 45° to the transmission axes of the crossed polarisers by rotating the sample until the bright state was located. In Fig. 3, the analyser was removed from the POM to enhance the contrast and appearance of the particle in the images.

### Disclination line control system

The control system was implemented in MATLAB and used both the Image Acquisition and Instrument Control Toolboxes. The BX51 optical microscope was configured as described above and frames from the CCD camera were analysed in a loop running at 10 iterations/frames per second. The position of the defect was determined by taking line-sections across the disclination line channel and using an in-built function findchangepts to find abrupt changes in a signal, corresponding to the position of the defect. This position is compared with a set position to produce an error value for each iteration of the loop. The voltage output from a Tektronix AFG3022 function generator producing a 1 kHz square wave was made proportional to this error signal. The voltage to hold the disclination line in place (when the two topologically discontinuous director states are of equal energy) was found experimentally. The control loop was configured with a bias so that the function generator outputs this experimentally-determined hold voltage when the error signal is equal to zero to avoid formation of the transient twisted (T) state. A GUI was created with a live view of the device in order to allow the control loop parameters to be tuned during operation and the set point to be adjusted by the user.

## Supplementary information

Supplementary Information

Description of Additional Supplementary Files

Supplementary Movie 1

Supplementary Movie 2

Supplementary Movie 3

Supplementary Movie 4

Supplementary Movie 5

Supplementary Movie 6

Supplementary Movie 7

## Data Availability

The datasets generated and analysed in this study are available on the Oxford Research Archive at https://dx.doi.org/10.5287/bodleian:2aarxBbd0.

## References

[1] Volovik, G. E. *Exotic Properties of Superfluid Helium 3*. (World Scientific Publishing Co Pte Ltd, 1992).

[2] Chaikin, P. & Lubensky, T. in *Principles of Condensed Matter Physics* 495–589 (Cambridge University Press, 1995).

[3] Moore JE (2010). The birth of topological insulators. Nature.

[4] Vilenkin, A. & Shellard, E. P. S. *Cosmic Strings and Other Topological Defects*. (Cambridge University Press, 2000).

[5] Chuang I, Durrer R, Turok N, Yurke B (1991). Cosmology in the laboratory: defect dynamics in liquid crystals. Science.

[6] Gennes, P. G. de. & Prost, J. The physics of liquid crystals. (Clarendon Press, 1993).

[7] Kleman M (1989). Defects in liquid crystals. Rep. Prog. Phys..

[8] Fleury JB, Pires D, Galerne Y (2009). Self-Connected 3D architecture of microwires. Phys. Rev. Lett..

[9] Blanc C, Coursault D, Lacaze E (2013). Ordering nano- and microparticles assemblies with liquid crystals. Liq. Cryst. Rev..

[10] Wang L, Li Q (2016). Stimuli-directing self-organized 3D liquid-crystalline nanostructures: from materials design to photonic applications. Adv. Funct. Mater..

[11] Li Y (2017). Periodic assembly of nanoparticle arrays in disclinations of cholesteric liquid crystals. Proc. Natl Acad. Sci. USA.

[12] Peng C, Turiv T, Guo Y, Wei QH, Lavrentovich OD (2016). Command of active matter by topological defects and patterns. Science.

[13] Liu Q (2010). Self-alignment of plasmonic gold nanorods in reconfigurable anisotropic fluids for tunable bulk metamaterial applications. Nano Lett..

[14] Senyuk B (2012). Shape-dependent oriented trapping and scaffolding of plasmonic nanoparticles by topological defects for self-assembly of colloidal dimers in liquid crystals. Nano Lett..

[15] Li, Q. *Liquid Crystals Beyond Displays: Chemistry, Physics and Applications*. (John Wiley & Sons, 2012).

[16] Ghadimi Nassiri M, Brasselet E (2018). Multispectral management of the photon orbital angular momentum. Phys. Rev. Lett..

[17] Marrucci L, Manzo C, Paparo D (2006). Optical spin-to-orbital angular momentum conversion in inhomogeneous anisotropic media. Phys. Rev. Lett..

[18] Brasselet E, Loussert C (2011). Electrically controlled topological defects in liquid crystals as tunable spin-orbit encoders for photons. Opt. Lett..

[19] Yoshida H, Asakura K, Fukuda J, Ozaki M (2015). Three-dimensional positioning and control of colloidal objects utilizing engineered liquid crystalline defect networks. Nat. Commun..

[20] Migara LK, Song J-K (2018). Standing wave-mediated molecular reorientation and spontaneous formation of tunable, concentric defect arrays in liquid crystal cells. NPG Asia Mater..

[21] Wang M, Li Y, Yokoyama H (2017). Artificial web of disclination lines in nematic liquid crystals. Nat. Commun..

[22] Malinauskas M, Farsari M, Piskarskas A, Juodkazis S (2013). Ultrafast laser nanostructuring of photopolymers: a decade of advances. Phys. Rep..

[23] Kawata S, Sun HB, Tanaka T, Takada K (2001). Finer features for functional microdevices. Nature.

[24] Soukoulis CM, Wegener M (2011). Past achievements and future challenges in the development of three-dimensional photonic metamaterials. Nat. Photonics.

[25] Gan Z, Cao Y, Evans RA, Gu M (2013). Three-dimensional deep sub-diffraction optical beam lithography with 9 nm feature size. Nat. Commun..

[26] Fischer J, Wegener M (2013). Three-dimensional optical laser lithography beyond the diffraction limit. Laser Photonics Rev..

[27] Tartan CC (2017). Generation of 3-dimensional polymer structures in liquid crystalline devices using direct laser writing. RSC Adv..

[28] Tartan CC (2018). Read on demand images in laser-written polymerizable liquid crystal devices. Adv. Opt. Mater..

[29] Bos PJ, Koehler/beran KR (1984). The pi-cell: a fast liquid-crystal optical-switching device. Mol. Cryst. Liq. Cryst..

[30] Nakamura H, Noguchi M (2000). Bend transition in Pi-cell.. Jpn. J. Appl. Phys..

[31] Acosta EJ, Towler MJ, Walton HG (2000). The role of surface tilt in the operation of pi-cell liquid crystal devices. Liq. Cryst..

[32] Jung J, Denniston C, Orlandini E, Yeomans JM (2003). Anisotropy of domain growth in nematic liquid crystals. Liq. Cryst..

[33] Smalyukh II, Lavrentovich OD, Kuzmin AN, Kachynski AV, Prasad PN (2005). Elasticity-mediated self-organization and colloidal interactions of solid spheres with tangential anchoring in a nematic liquid crystal. Phys. Rev. Lett..

[34] Sasaki Y (2016). Large-scale self-organization of reconfigurable topological defect networks in nematic liquid crystals. Nat. Commun..

[35] Bisoyi HK, Kumar S (2011). Liquid-crystal nanoscience: an emerging avenue of soft self-assembly. Chem. Soc. Rev..

[36] Lavrentovich OD (2014). Transport of particles in liquid crystals. Soft Matter.

[37] Kikuchi H (2005). Bend-mode liquid crystal cells stabilized by aligned polymer walls. Jpn. J. Appl. Phys..

