# Supplementary material for: Electrically-tunable positioning of topological defects in liquid crystals
Source: Nat Commun. 2020 May 5;11:2203. doi: 10.1038/s41467-020-16059-1 (PMC7200663; doi:10.1038/s41467-020-16059-1)
Supplement: Supplementary file 1 — Supplementary Information [file 41467_2020_16059_MOESM1_ESM.pdf]

Supplementary Information

Electrically-Tunable Positioning of Topological Defects in Liquid Crystals

John J. Sandford O'Neill, Patrick S. Salter, Martin J. Booth, Steve J. Elston, Stephen M. Morris

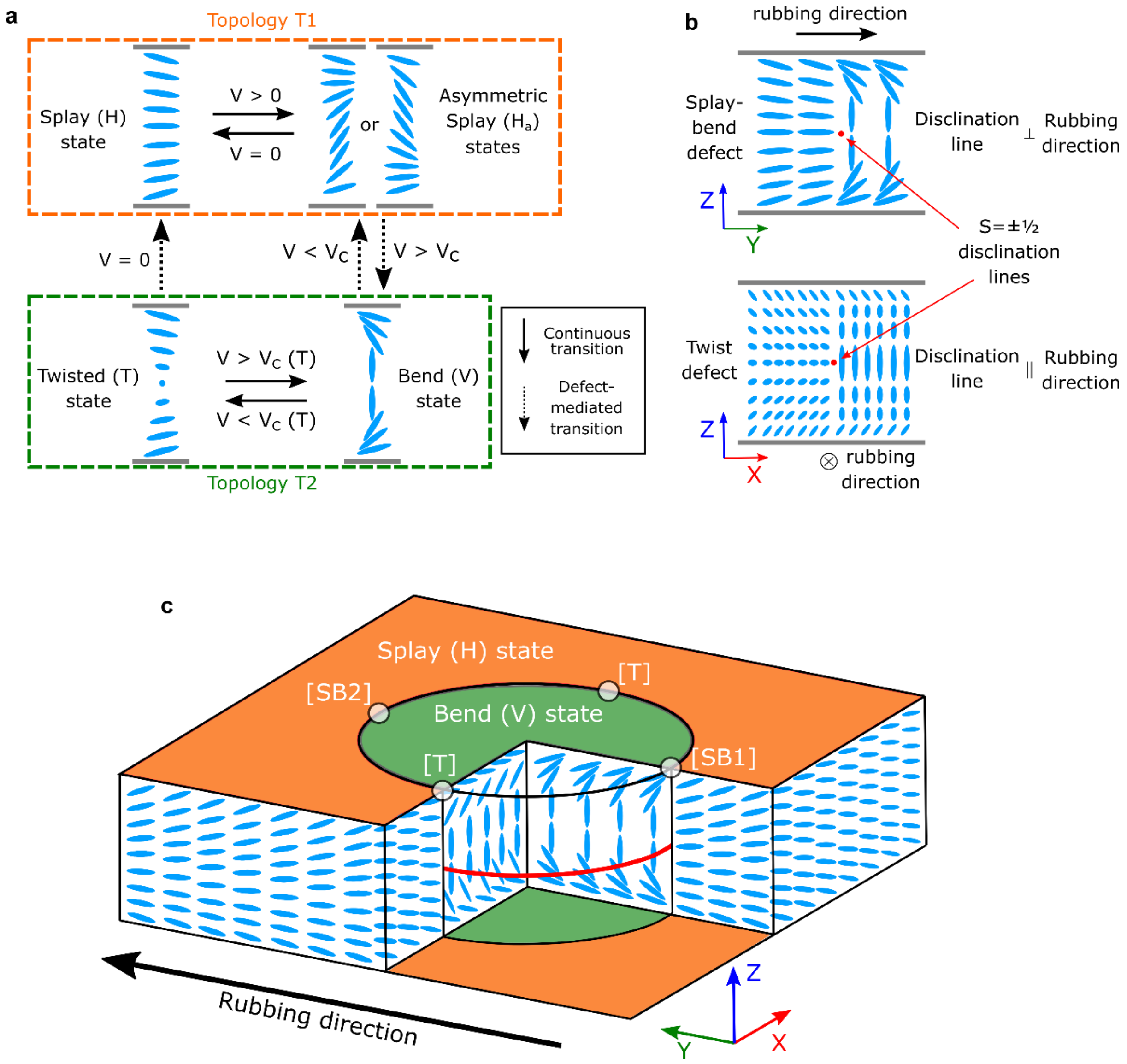

Supplementary Figure 1: Voltage-dependent states of the pi-cell. (a) Director profiles of the different director states possible in LC pi-cells. Blue rods show the orientation of liquid crystal molecules. The ground state under zero applied voltage is the splay (H) state, where the LC director lies in the plane of the device substrates along the rubbing direction. Applying a voltage causes the H state to distort into one of two asymmetric splay (H_a) states. These are optically identical and topologically continuous but can be distinguished by the thread-like domain wall that forms on the boundary between the two states when viewed by polarising optical microscopy. If a voltage is applied suddenly to the device, the states form randomly across the cell due to the random thermal fluctuations of the LC director at the instant the voltage switch-on occurs (example in Supplementary Fig. 2). However, if a voltage-ramp is applied to the cell, there will be parts of the device where the same H_a state always forms, due to slight asymmetries in the pre-tilt angle between the two substrates. Applying a voltage above the critical voltage, V_c, causes the formation of the bend (V) state that is topologically distinct from the H-states. This transition is slow and occurs via defect-mediated domain growth in the device. V-state domains will grow, bounded by strength ±½ disclination loops that separate the growing V-domains from the retreating H-state domains. Once a device has transformed into the V state, decreasing the voltage below V_c reverses the transition and causes the splay (H) state to become the lowest energy state and slowly propagate across the device in a similar manner.

Decreasing the voltage further, below another critical voltage (V_c (T)) causes the V state domains to collapse into a 180° transient twisted (T) state. The T state is topologically continuous with the V state but discontinuous with the H state. As V_c (T) < V_c, the T state is transient and will eventually be consumed by the slow defect-mediated transition back to the H state. There will always be a defect on the boundary between states with topology T1 and a state with topology T2. (b) 2D director profiles showing the different types of defect that exist in LC pi cells. Splay-bend type defects, where the distortion surrounding the defect is of a splay and bend nature, occur when the disclination line is perpendicular to the rubbing direction of the device. Conversely, twist type defects occur when the disclination line is parallel to the rubbing direction and the defect has a twisted nature. There is a lower elastic energy cost associated with twist deformations in LCs as compared to splay and bend type deformations and thus the twist defect has a lower elastic energy and therefore a higher stability than splay-bend defects. In reality this means that splay-bend defects have a tendency to distort, forming zig-zag structures to become more parallel to the rubbing direction, such that the defect obtains a partially twisted nature and lowers its elastic energy (see Supplementary Fig. 5). (c) 3D schematic director profile of a V state domain surrounded by an H state domain. The red line represents the defect loop separating the two topologically discontinuous domains. The rubbing direction is along the Y-axis and the 2D projections in (b) correspond to the same geometry. The nature of the disclination varies along the length of the defect loop: at the positions marked SB1 and SB2 the defect has a completely splay-bend nature whereas at the points marked T the defect has a completely twisted nature. Adapted from Jung, J., Denniston, C., Orlandini, E. & Yeomans, J. M. Anisotropy of domain growth in nematic liquid crystals. *Liquid Crystals* **30**, 1455–1462 (2003) with permission of Taylor & Francis Ltd. http://www.tandfonline.com.

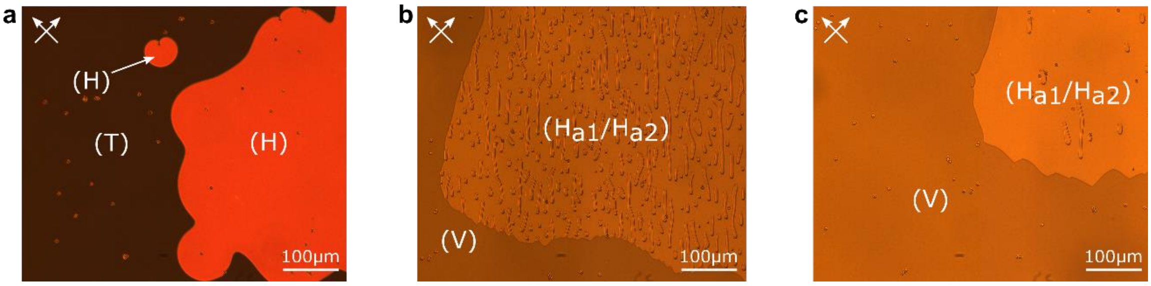

Supplementary Figure 2: Random nucleation of defects in conventional LC pi-cells. Defect formation and the nucleation of topologically discontinuous director states in a conventional LC pi-cell without any fabricated polymer structures. (a) Random nucleation of the H state at zero applied voltage. The device was initially driven into the V state by applying a voltage $V > V_c$, before the voltage was removed to allow the cell to relax to its ground state. Nucleation of the H state occurs randomly and can be seen to originate from the spacer beads in the cell, which cause a strong distortion of the director around them. (b) Growth of the V state and transition into the asymmetric H states. A voltage, $V > V_c$ is applied to the cell, allowing domain growth of the V state to occur randomly. The disclination line between the V state and the H states has an irregular morphology that depends on where the V state randomly nucleated in the cell. The H state region transitions into a mixture of the two asymmetric H states, with thread-like loops marking the boundaries between the H_a states. (c) The V state domain has grown, and the asymmetric H-state region has largely collapsed into one of the two asymmetric H states via H_{a1}/H_{a2} domain wall annihilation.

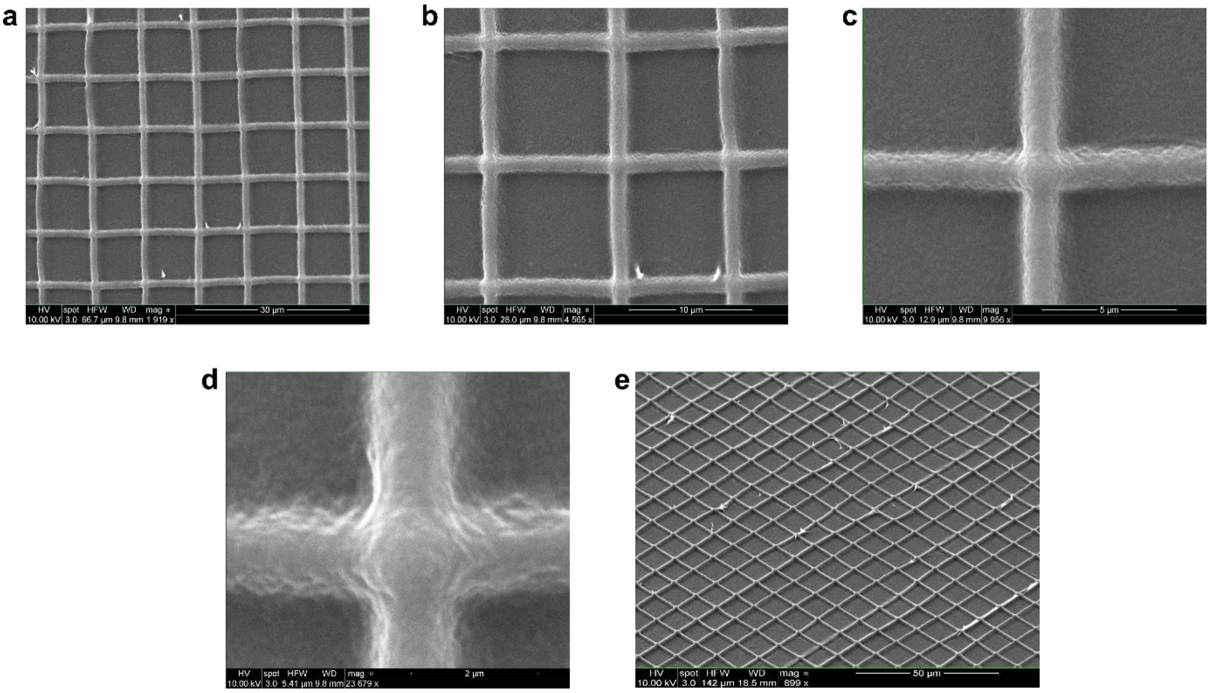

Supplementary Figure 3: Scanning electron microscopy (SEM) images of polymer structures fabricated with 2PP-DLW. To visualise the topography of the polymer features fabricated by 2PP-DLW, SEM images were taken of exemplar devices fabricated in analogous conditions to those presented in the main text. (a)-(d) show images of a 2PP-DLW square lattice with 10 µm period, fabricated in a 5 µm thick LC device containing the same polymerizable mixture used elsewhere in this study. The cell was then immersed in acetone for 24 hours and disassembled. The substrate to which the polymer network was attached was coated with a layer of gold approximately 20 nm thick before being loaded into a FEI Quanta 600 FEG scanning electron microscope and imaged with the secondary electron detector. All images were taken with an electron beam voltage of 10 kV and images (a-d) were taken at a working distance of 9.8 mm. Image (e) was taken by tilting the sample by 45° with respect to the electron beam at a working distance of 18.5 mm. It can be seen that the polymer walls are typically 1 µm in width, in close agreement with the theoretically expected spot-size of the fabrication laser ($w = 0.61\lambda/NA = 0.96$ µm). Therefore we can assume that there is only a limited amount of diffusion of the reactive mesogen beyond the irradiated regions. We note that the features fabricated in this study by 2PP-DLW are designed to control liquid crystal alignment, as opposed to acting as free form polymeric structures, and are therefore expected to display greater porosity than in typical 2PP. Thus, there is likely some degradation of the polymer network during the washing out, cell deconstruction and gold coating processes, which can account for non-uniformity seen in the images.

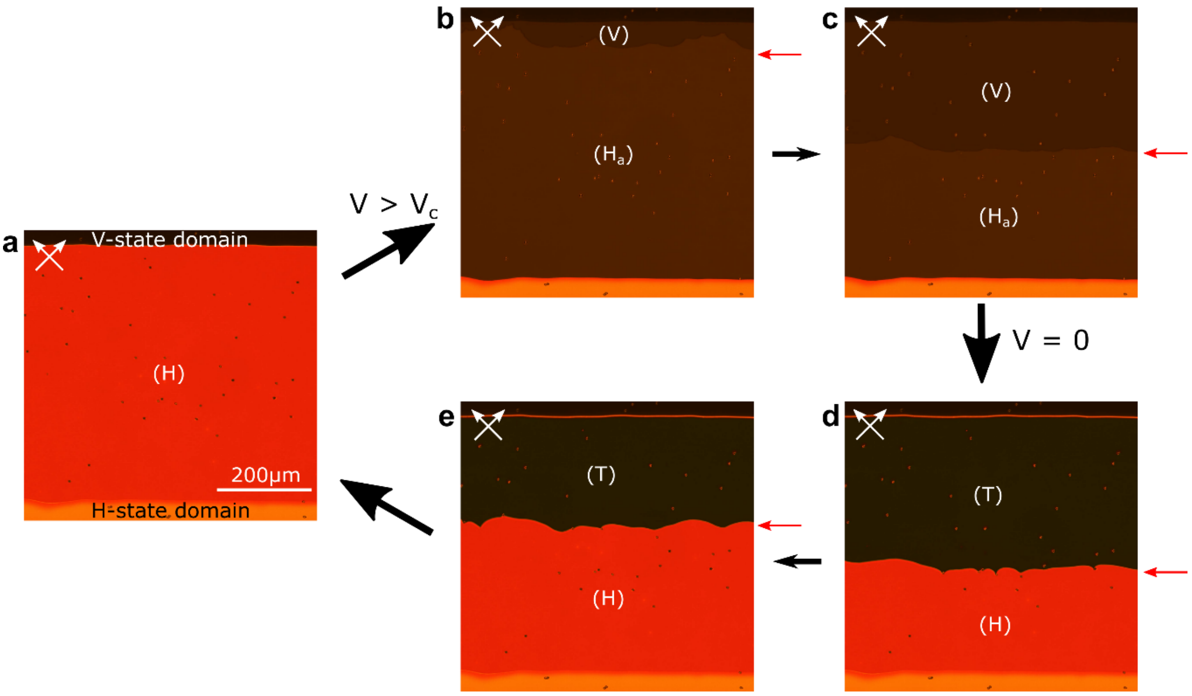

Supplementary Figure 4: Polarising Optical Microscopy images of a tunable disclination line fabricated with UV photolithography. To demonstrate other fabrication methods to generate tunable defect states, the V- and H-state domains were also stabilised in a pi-cell device using single photon polymerization with a 365 nm UV LED. Each region was sequentially polymerised with an intensity of 0.3 mW/cm^2 for 30 seconds, using a series of photomasks placed on the outer surface of the cell's glass substrates to create an uncured channel approximately 540 µm wide. The red arrows indicate the position of the defect. (a) the structure with no applied voltage. (b) a 6 V ramp over 5 s applied to the cell results in the nucleation and growth of the V state and (c) the movement of the defect across the cell. (d) the voltage is switched-off, causing the H state to grow and the V-state to collapse to the T-state. (e) the defect moves back across the cell in the absence of an applied voltage towards the V-state domain. The behaviour of the defect line in response to an applied voltage is broadly similar to that created with 2PP-DLW, however, the nucleation of the V state on the boundary of the V-state polymer region and movement of the defect is less uniform. Furthermore, it is more-challenging to create fine features due to the need to project an image of the photomask into the cell and accurately register a series of photomasks.

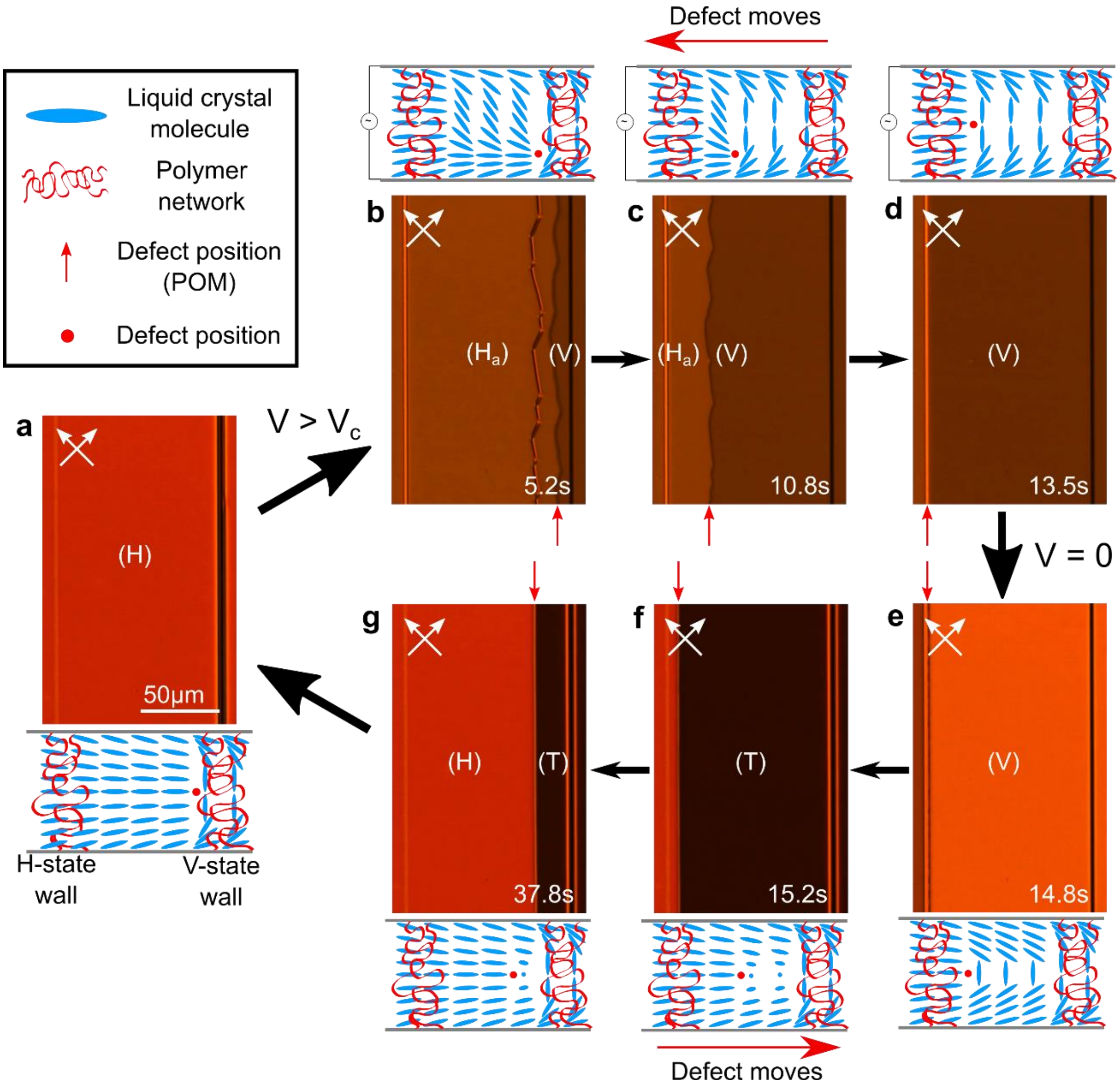

Supplementary Figure 5: Tunable disclination line written perpendicular to the device rubbing direction. Polarising optical microscopy images and director profiles of the dynamics of a tunable defect channel under a linear voltage ramp up to 4.5 V over a period of 5s. The polymer walls are written perpendicular to the device rubbing direction (therefore the defect has a splay-bend nature) and the director profiles illustrated correspond to this case. Twist-type distortions, that occur when the disclination line is parallel to the rubbing direction, have a lower elastic energy. Thus splay-bend type defects will distort, forming a zig-zag morphology to increase their twisted nature. (a) With no voltage applied, the bulk of the device is in the splay (H) state and a defect has formed adjacent to the polymer-stabilised bend (V) state wall. A voltage above V_c is applied to the device, so that the bend state now has a lower free-energy density. (b) The bend-state nucleates from the wall, (c) moving the defect separating the topologically discontinuous states across the channel (d) until it meets the polymer stabilised splay state wall. (e) Removing the voltage causes the bend state to relax into a 180° twisted state (T-state), and (f) the splay state to nucleate from the splay state wall. (g) The splay state grows and moves across the channel with the disclination line at the frontier of the advancing domain until it is impeded by the polymer-stabilised bend state wall. All images are between crossed polarisers with the device rubbing direction oriented 45° to the transmission axes of the polarisers.

There is additional complexity observed close to the bend state polymer wall as the voltage is applied (Supplementary Fig. 5b). In pi-cells there are two possible director states that occur when a voltage is applied to the splay state that are known as the asymmetric H-states (Supplementary Fig. 1a). The tunable disclination line was fabricated in a region of the device where only one of these states forms. However, we can observe in Supplementary Fig. 5b that the disclination line causes the opposite asymmetric H-state to form in the vicinity of the bend state polymer wall and so a domain boundary between the two asymmetric H states with a zig-zag deformation can be seen near the polymer wall.

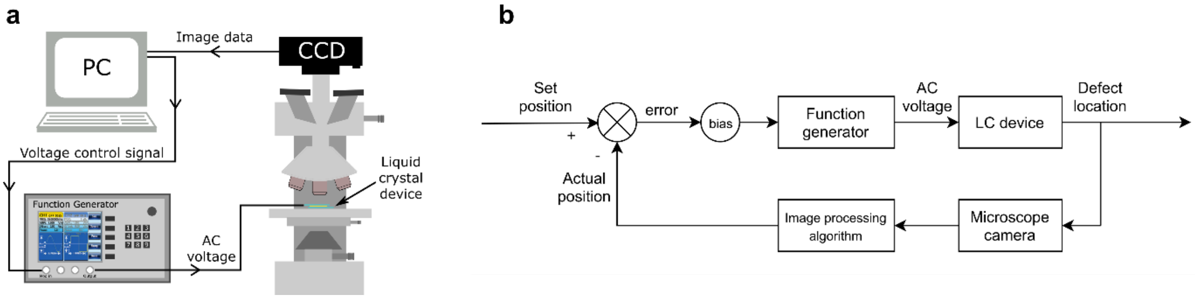

Supplementary Figure 6: Tunable disclination line control system. (a) Schematic diagram of the elements of the control system. The microscope CCD sends images of the device to the control system program running in MATLAB. The program adjusts the voltage applied to the device via the function generator in order to move the defect to the set position specified by the user. (b) Control loop block diagram. The position of the defect is detected with an image recognition algorithm and compared with the set position. This voltage output by a function generator connected to the device is made proportional to this error signal in order to control the position of the defect.

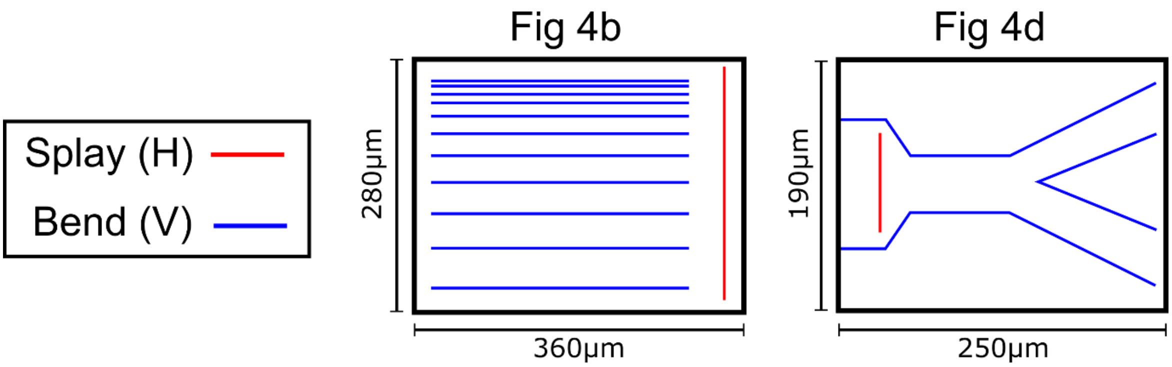

Supplementary Figure 7: Schematic designs of complex polymer structures for disclination line control. Schematic illustrations of the polymer wall designs for two structures: defect-confinement channels shown in Fig. 4b and the bifurcated defect channel shown in Fig. 4d.
