# Supplementary material for: Electrically-tunable positioning of topological defects in liquid crystals
Source: Nat Commun. 2020 May 5;11:2203. doi: 10.1038/s41467-020-16059-1 (PMC7200663; doi:10.1038/s41467-020-16059-1)
Supplement: Supplementary file 2 — Description of Additional Supplementary Files [file 41467_2020_16059_MOESM2_ESM.pdf]

Description of Additional Supplementary Files

File Name: Supplementary Movie 1
Description: This video shows the real-time dynamic behaviour of the tunable disclination line structure shown in Fig. 1. The polymer walls at the top and bottom of the frame were written in the bend (V) state and the splay (H) state respectively. The polymer walls and disclination line were parallel to the rubbing direction of the device, meaning that the defect between the two states is of a twist type. The device is driven with a linear voltage ramp from 0 V to 4.5 V over a period of 5 seconds and held at 4.5 V until the defect reaches the splay state wall. The voltage is then switched-off, whereupon the defect reverses direction and returns to the bend state wall. The defect has a stable morphology as a twist-type defect has a lower elastic energy cost than a splay-bend type defect.

File Name: Supplementary Movie 2
Description: This video shows the real-time dynamic behaviour of the tunable disclination line structure shown in Supplementary Fig. 5. The polymer walls at the top and bottom of the frame were written in the bend (V) state and the splay (H) state respectively. The walls were perpendicular to the rubbing direction of the device, meaning that the defect between the two states is a splay-bend type defect. The device is driven with a linear voltage ramp from 0 V to 4.5 V over a period of 5 seconds and held at 4.5 V until the defect reaches the splay state wall. The voltage is then switched-off, whereupon the defect reverses direction and returns to the bend state wall. The defect has an unstable zig-zag morphology as a splay-bend type defect has a higher elastic energy cost than a twist type defect, so the defect line lengthens and distorts into a zigzag shape to give it a partially twisted nature.

File Name: Supplementary Movie 3
Description: This video shows the real-time operation of the control loop shown and described in Fig. 2. The control loop regulates the tunable disclination line structure shown in Fig. 1 and Supplementary Movie 1. The set position is indicated by the dashed blue line and the current detected position of the defect is indicated by the red cross. The series of changes in the set position shown in this video correspond the graph shown in Fig. 2c.

File Name: Supplementary Movie 4
Description: This is a real-time video of the polarising optical micrographs shown in Fig. 3 demonstrating microparticle transport using a tunable disclination line. The polymer wall on the left-hand side was written in the splay state and the polymer wall on the right-hand side was written in the bend state and the walls are separated by 100 µm. Initially the device has been driven to the bend state with the application of a voltage above V_c. At $t = 0.3$ s the voltage is switched off and the defect moves from the left to the right. When it meets the 1 µm diameter silica microsphere, the defect line traps the particle, transporting it across the channel to the other side. The analyser was removed from the POM to enhance the image.

File Name: Supplementary Movie 5
Description: Real-time video showing the behaviour of the circular defect rings shown in Fig. 4a. At the start of the movie the device is driven in the V-state and two circular polymer walls can be seen that were written in the splay (H) state at 0 V. The voltage is switched off at $t = 1.3$ s, which causes

the bend state region to collapse to the twisted (T) state and allows the splay (H) ground state to grow, as it is the lowest energy state at zero volts. The disclination lines emanating from the polymer rings that separate the H state from the T state expand in a circular fashion as the device relaxes. When the two circular defects meet, they annihilate and form a contiguous splay-state region.

File Name: Supplementary Movie 6
Description: Real-time video showing the behaviour of the defect-confinement channels shown in the micrographs in Fig. 4b. The device is initially in the bend state and the voltage is switched off at t = 4.6 s. The defect next to the splay-state wall on the right-hand side of the frame then moves from right to left. When it reaches the parallel bend-state walls the defect slows down. The degree to which it slows down depends on the width of the confinement channels. A design of the structure can be seen in Supplementary Fig. 7.

File Name: Supplementary Movie 7
Description: Real-time video showing the behaviour of the bifurcated channel shown in Fig. 4d. The device is initially driven into the bend state with an applied voltage above Vc before the voltage is switched off at t = 8.6 s. The defect then grows from the splay-state polymer wall towards the left of the frame and begins to move down the channel, the walls of which were written in the bend-state. A design of the structure can be seen in Supplementary Fig. 7. When the defect line reaches the fork it is forced to split and travel down the two smaller channels.
